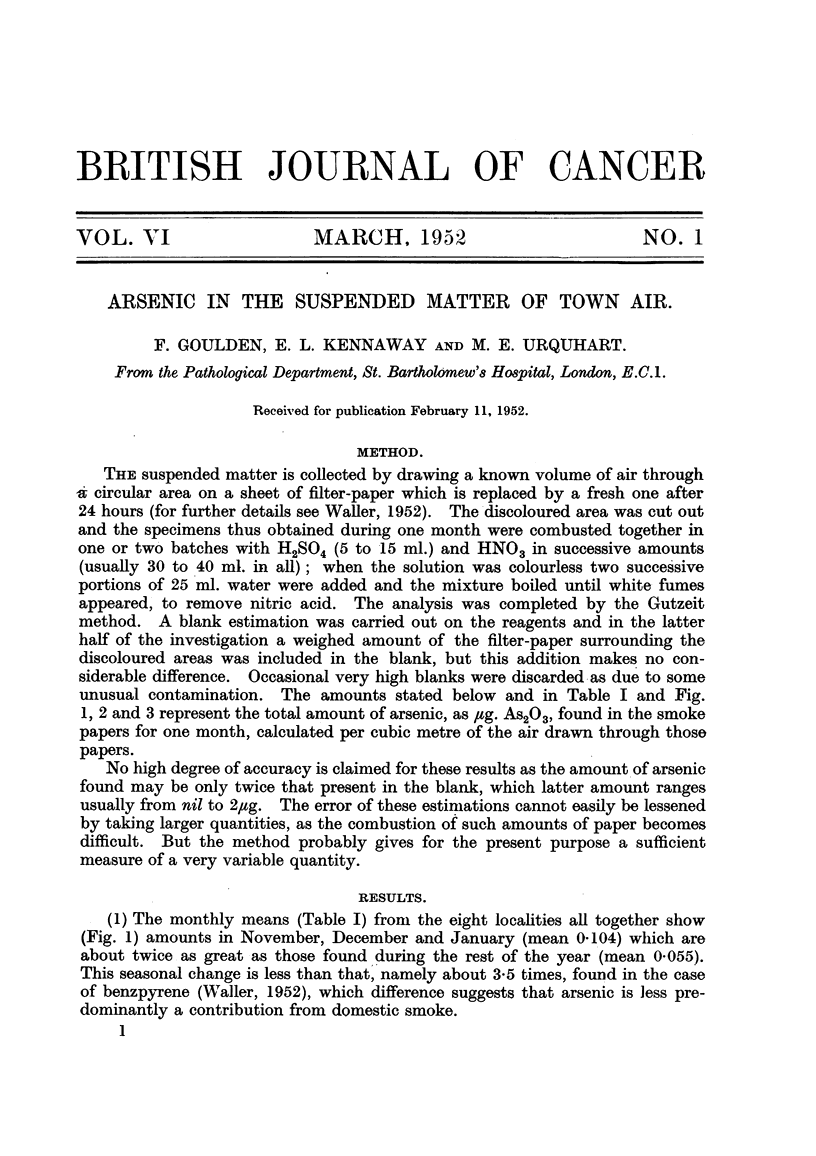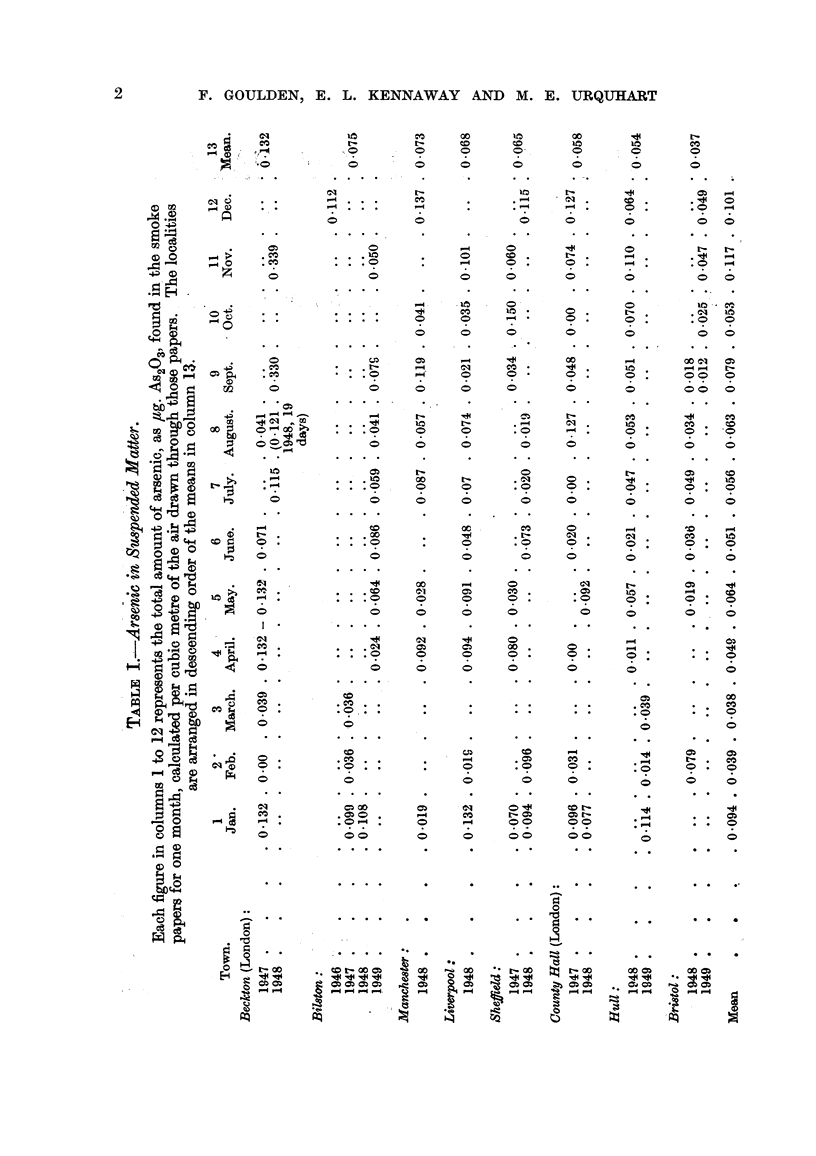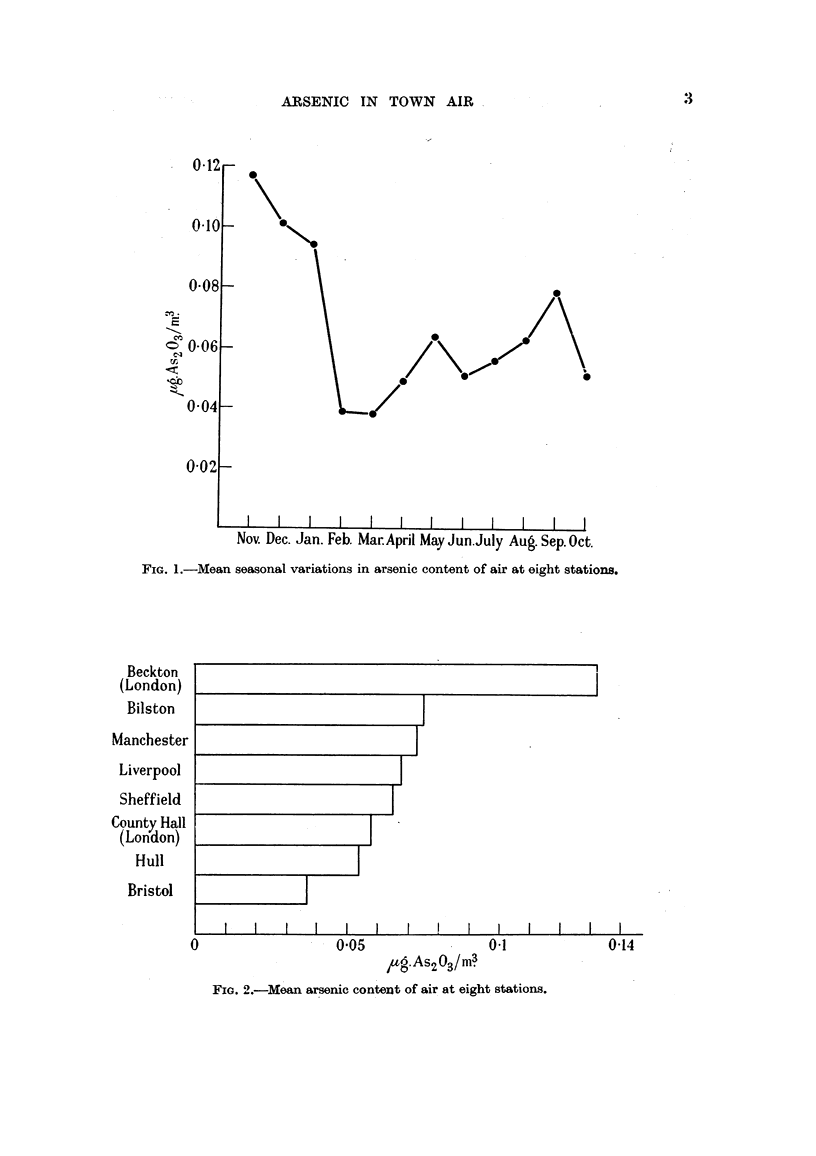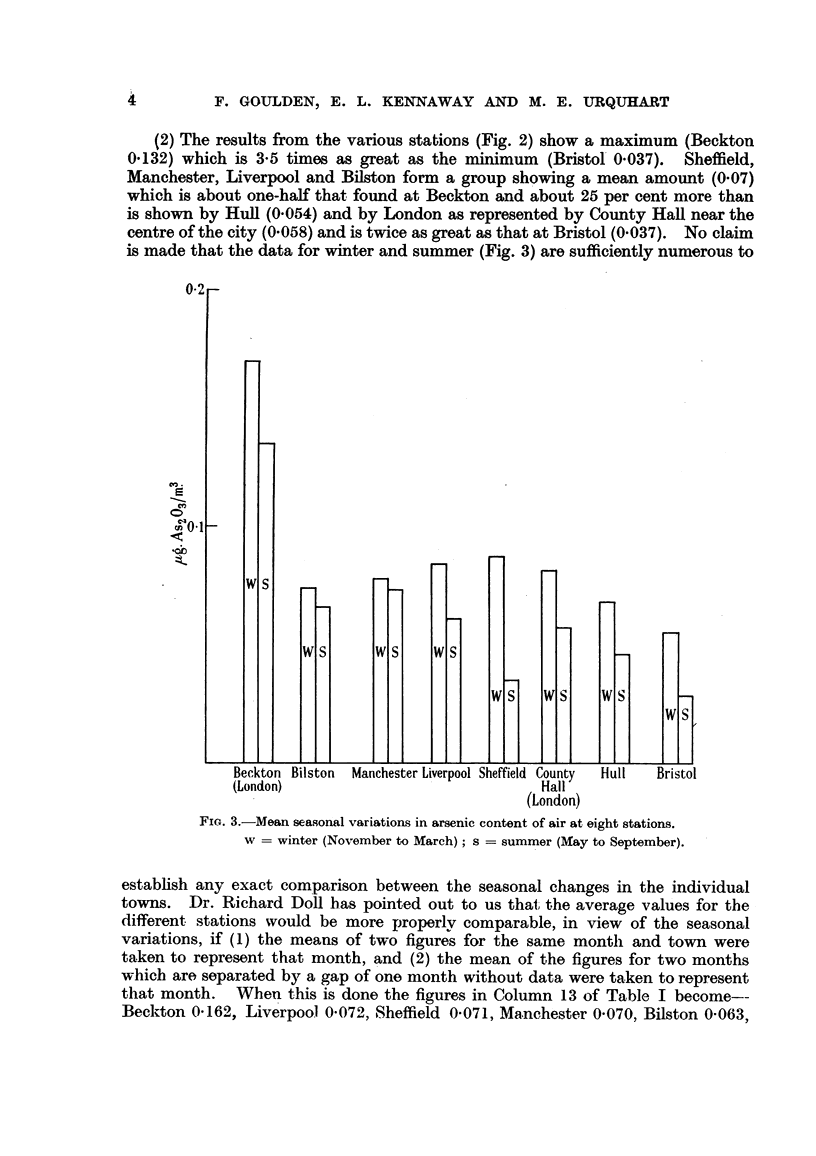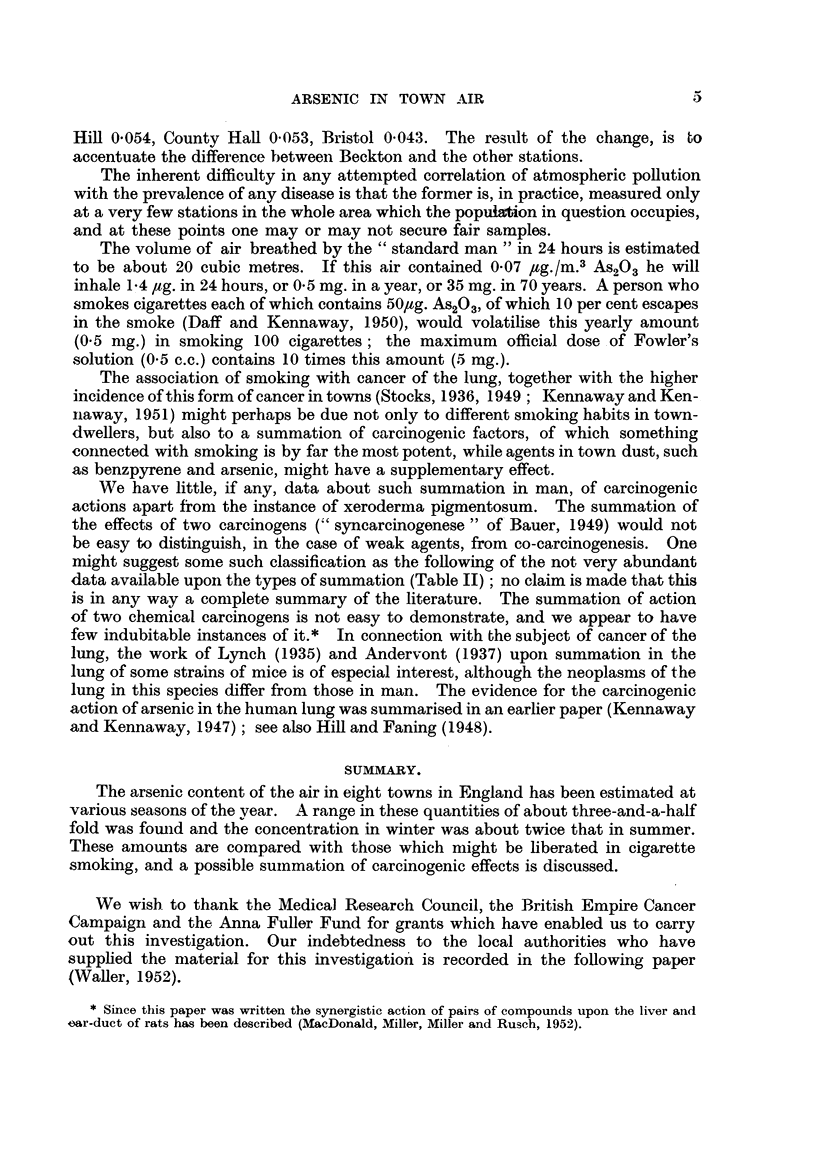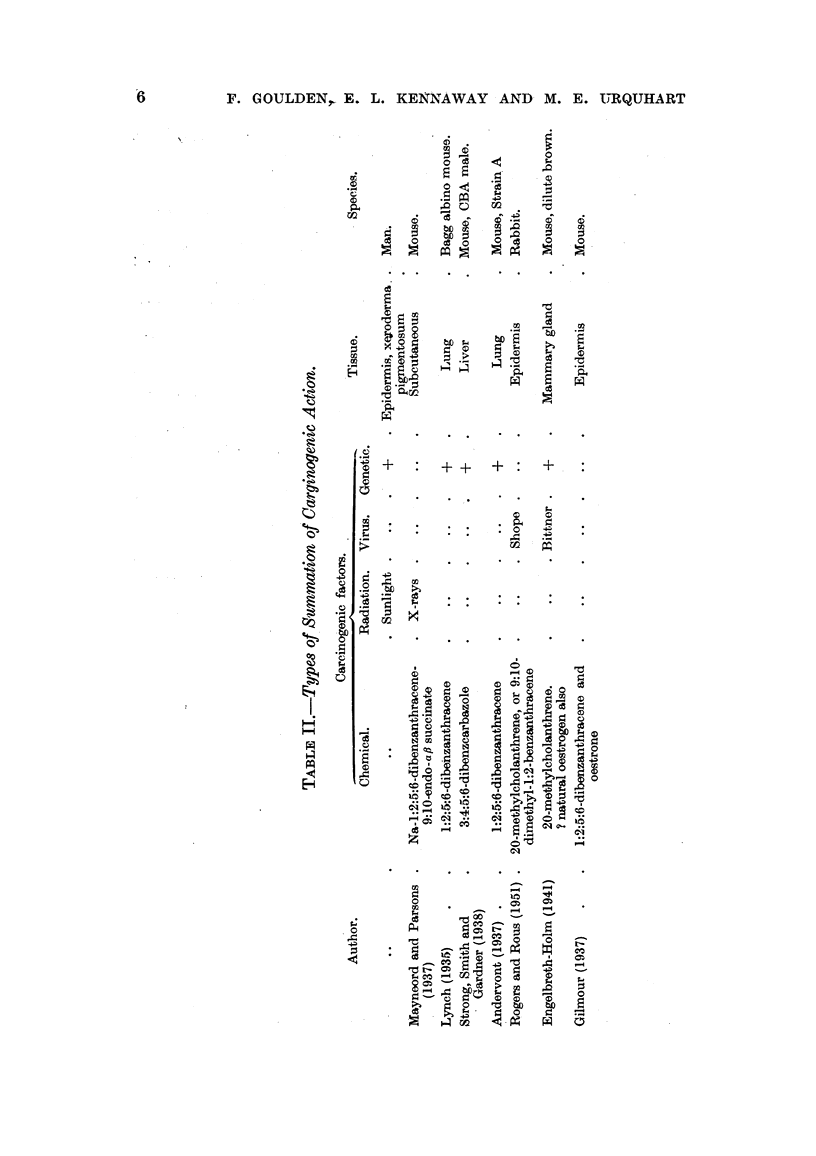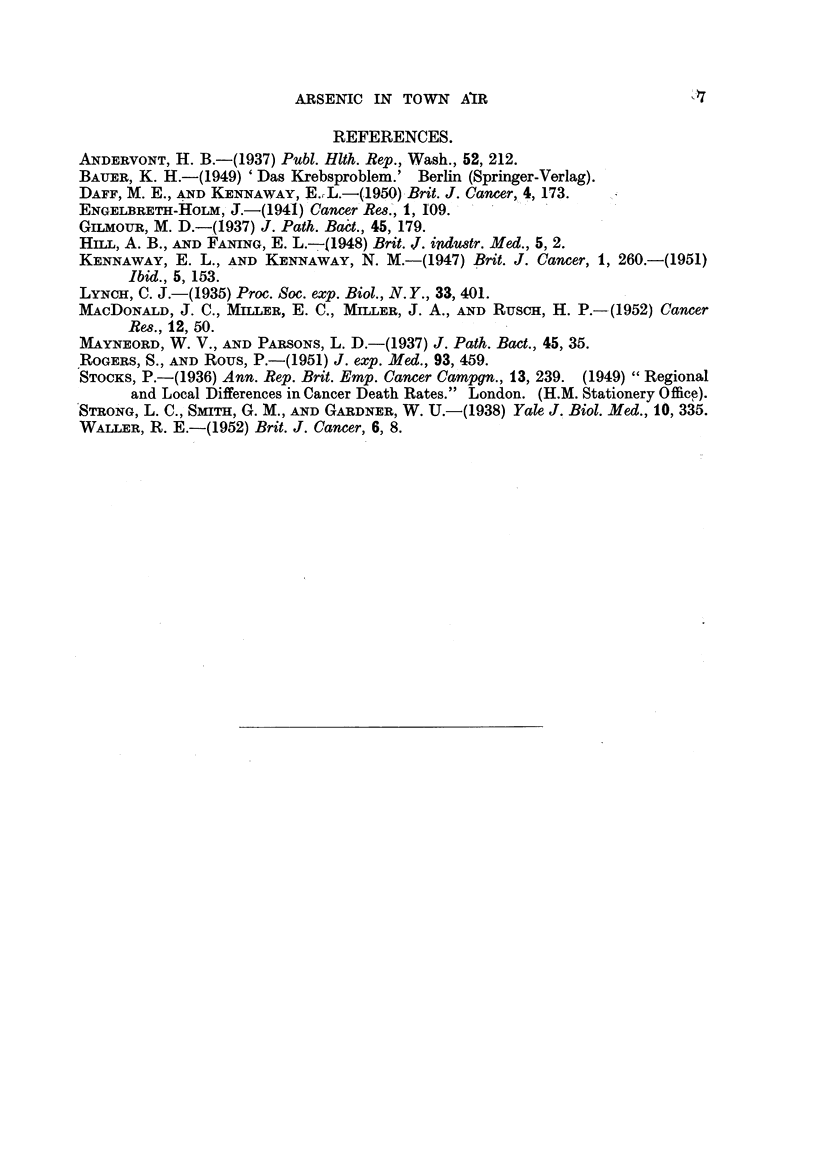# Arsenic in the Suspended Matter of Town Air

**DOI:** 10.1038/bjc.1952.1

**Published:** 1952-03

**Authors:** F. Goulden, E. L. Kennaway, M. E. Urquhart


BRITISH JOURNAL OF CANCER

VOL. VI | MARCH, 1952 | NO. 1

### ARSENIC IN THE SUSPENDED MATTER OF TOWN AIR.

F. GOULDEN, E. L. KENNAWAY AND M. E. URQUHART.

*From the Pathological Department, St. Bartholomew's Hospital, London, E.C.*1.

Received for publication February 11, 1952.

### METHOD.

THE suspended matter is collected by drawing a known volume of air through a circular area on a sheet of filter-paper which is replaced by a fresh one after 24 hours (for further details see Waller, 1952). The discoloured area was cut out and the specimens thus obtained during one month were combusted together in one or two batches with $H_2SO_4$ (5 to 15 ml.) and $HNO_3$ in successive amounts (usually 30 to 40 ml. in all); when the solution was colourless two successive portions of 25 ml. water were added and the mixture boiled until white fumes appeared, to remove nitric acid. The analysis was completed by the Gutzeit method. A blank estimation was carried out on the reagents and in the latter half of the investigation a weighed amount of the filter-paper surrounding the discoloured areas was included in the blank, but this addition makes no considerable difference. Occasional very high blanks were discarded as due to some unusual contamination. The amounts stated below and in Table I and Fig. 1, 2 and 3 represent the total amount of arsenic, as $\mu g.$ $As_2O_3$, found in the smoke papers for one month, calculated per cubic metre of the air drawn through those papers.

No high degree of accuracy is claimed for these results as the amount of arsenic found may be only twice that present in the blank, which latter amount ranges usually from *nil* to $2 \mu g.$ The error of these estimations cannot easily be lessened by taking larger quantities, as the combustion of such amounts of paper becomes difficult. But the method probably gives for the present purpose a sufficient measure of a very variable quantity.

### RESULTS.

(1) The monthly means (Table I) from the eight localities all together show (Fig. 1) amounts in November, December and January (mean 0·104) which are about twice as great as those found during the rest of the year (mean 0·055). This seasonal change is less than that, namely about 3·5 times, found in the case of benzpyrene (Waller, 1952), which difference suggests that arsenic is less predominantly a contribution from domestic smoke.

### Table I.—*Arsenic in Suspended Matter.*

FIG. 1.—Mean seasonal variations in arsenic content of air at eight stations.

FIG. 2.—Mean arsenic content of air at eight stations.

(2) The results from the various stations (Fig. 2) show a maximum (Beckton 0·132) which is 3·5 times as great as the minimum (Bristol 0·037). Sheffield, Manchester, Liverpool and Bilston form a group showing a mean amount (0·07) which is about one-half that found at Beckton and about 25 per cent more than is shown by Hull (0·054) and by London as represented by County Hall near the centre of the city (0·058) and is twice as great as that at Bristol (0·037). No claim is made that the data for winter and summer (Fig. 3) are sufficiently numerous to

FIG. 3.—Mean seasonal variations in arsenic content of air at eight stations. W = winter (November to March); S = summer (May to September).

establish any exact comparison between the seasonal changes in the individual towns. Dr. Richard Doll has pointed out to us that the average values for the different stations would be more properly comparable, in view of the seasonal variations, if (1) the means of two figures for the same month and town were taken to represent that month, and (2) the mean of the figures for two months which are separated by a gap of one month without data were taken to represent that month. When this is done the figures in Column 13 of Table I become—Beckton 0·162, Liverpool 0·072, Sheffield 0·071, Manchester 0·070, Bilston 0·063,

Hill 0·054, County Hall 0·053, Bristol 0·043. The result of the change, is to accentuate the difference between Beckton and the other stations.

The inherent difficulty in any attempted correlation of atmospheric pollution with the prevalence of any disease is that the former is, in practice, measured only at a very few stations in the whole area which the population in question occupies, and at these points one may or may not secure fair samples.

The volume of air breathed by the " standard man " in 24 hours is estimated to be about 20 cubic metres. If this air contained 0·07 $\mu$g./m.$^3$ $As_2O_3$ he will inhale 1·4 $\mu$g. in 24 hours, or 0·5 mg. in a year, or 35 mg. in 70 years. A person who smokes cigarettes each of which contains 50$\mu$g. $As_2O_3$, of which 10 per cent escapes in the smoke (Daff and Kennaway, 1950), would volatilise this yearly amount (0·5 mg.) in smoking 100 cigarettes ; the maximum official dose of Fowler's solution (0·5 c.c.) contains 10 times this amount (5 mg.).

The association of smoking with cancer of the lung, together with the higher incidence of this form of cancer in towns (Stocks, 1936, 1949 ; Kennaway and Kennaway, 1951) might perhaps be due not only to different smoking habits in town-dwellers, but also to a summation of carcinogenic factors, of which something connected with smoking is by far the most potent, while agents in town dust, such as benzpyrene and arsenic, might have a supplementary effect.

We have little, if any, data about such summation in man, of carcinogenic actions apart from the instance of xeroderma pigmentosum. The summation of the effects of two carcinogens (" syncarcinogenese " of Bauer, 1949) would not be easy to distinguish, in the case of weak agents, from co-carcinogenesis. One might suggest some such classification as the following of the not very abundant data available upon the types of summation (Table II) ; no claim is made that this is in any way a complete summary of the literature. The summation of action of two chemical carcinogens is not easy to demonstrate, and we appear to have few indubitable instances of it.* In connection with the subject of cancer of the lung, the work of Lynch (1935) and Andervont (1937) upon summation in the lung of some strains of mice is of especial interest, although the neoplasms of the lung in this species differ from those in man. The evidence for the carcinogenic action of arsenic in the human lung was summarised in an earlier paper (Kennaway and Kennaway, 1947) ; see also Hill and Faning (1948).

### SUMMARY.

The arsenic content of the air in eight towns in England has been estimated at various seasons of the year. A range in these quantities of about three-and-a-half fold was found and the concentration in winter was about twice that in summer. These amounts are compared with those which might be liberated in cigarette smoking, and a possible summation of carcinogenic effects is discussed.

We wish to thank the Medical Research Council, the British Empire Cancer Campaign and the Anna Fuller Fund for grants which have enabled us to carry out this investigation. Our indebtedness to the local authorities who have supplied the material for this investigation is recorded in the following paper (Waller, 1952).

* Since this paper was written the synergistic action of pairs of compounds upon the liver and ear-duct of rats has been described (MacDonald, Miller, Miller and Rusch, 1952).

### REFERENCES.

Andervont, H. B.—(1937) *Publ. Hlth. Rep.*, Wash., **52**, 212.
Bauer, K. H.—(1949) 'Das Krebsproblem.' Berlin (Springer-Verlag).
Daff, M. E., and Kennaway, E. L.—(1950) *Brit. J. Cancer*, **4**, 173.
Engelbreth-Holm, J.—(1941) *Cancer Res.*, **1**, 109.
Gilmour, M. D.—(1937) *J. Path. Bact.*, **45**, 179.
Hill, A. B., and Faning, E. L.—(1948) *Brit. J. industr. Med.*, **5**, 2.
Kennaway, E. L., and Kennaway, N. M.—(1947) *Brit. J. Cancer*, **1**, 260.—(1951) *Ibid.*, **5**, 153.
Lynch, C. J.—(1935) *Proc. Soc. exp. Biol., N.Y.*, **33**, 401.
MacDonald, J. C., Miller, E. C., Miller, J. A., and Rusch, H. P.—(1952) *Cancer Res.*, **12**, 50.
Mayneord, W. V., and Parsons, L. D.—(1937) *J. Path. Bact.*, **45**, 35.
Rogers, S., and Rous, P.—(1951) *J. exp. Med.*, **93**, 459.
Stocks, P.—(1936) *Ann. Rep. Brit. Emp. Cancer Campgn.*, **13**, 239. (1949) "Regional and Local Differences in Cancer Death Rates." London. (H.M. Stationery Office).
Strong, L. C., Smith, G. M., and Gardner, W. U.—(1938) *Yale J. Biol. Med.*, **10**, 335.
Waller, R. E.—(1952) *Brit. J. Cancer*, **6**, 8.